# Development of a Fully Protective Pandemic Avian Influenza Subunit Vaccine in Insect Pupae

**DOI:** 10.3390/v16060829

**Published:** 2024-05-23

**Authors:** Ana Falcón, Susana Martínez-Pulgarín, Sergi López-Serrano, Edel Reytor, Miguel Cid, Maria del Carmen Nuñez, Lorena Córdoba, Ayub Darji, José M. Escribano

**Affiliations:** 1 Alternative Gene Expression S.L. (ALGENEX), Ronda de Poniente 14, 28760 Madrid, Spain; 2 Unitat Mixta d’Investigació IRTA-UAB en Sanitat Animal, Centre de Recerca en Sanitat Animal (CReSA), Campus de la Universitat Autònoma de Barcelona (UAB), 08193 Bellaterra, Spain; 3 Programa de Sanitat Animal, Institut de Recerca i Tecnologia Agroalimentàries, Centre de Recerca en Sanitat Animal (CReSA), Campus de la Universitat Autònoma de Barcelona (UAB), 08193 Bellaterra, Spain

**Keywords:** pandemic influenza, baculovirus, *Trichoplusia ni*, insect pupae, subunit vaccine

## Abstract

In this study, we pioneered an alternative technology for manufacturing subunit influenza hemagglutinin (HA)-based vaccines. This innovative method involves harnessing the pupae of the Lepidoptera *Trichoplusia ni* (*T. ni*) as natural biofactories in combination with baculovirus vectors (using CrisBio^®^ technology). We engineered recombinant baculoviruses encoding two versions of the HA protein (trimeric or monomeric) derived from a pandemic avian H7N1 virus A strain (A/chicken/Italy/5093/99). These were then used to infect *T. ni* pupae, resulting in the production of the desired recombinant antigens. The obtained HA proteins were purified using affinity chromatography, consistently yielding approximately 75 mg/L of insect extract. The vaccine antigen effectively immunized poultry, which were subsequently challenged with a virulent H7N1 avian influenza virus. Following infection, all vaccinated animals survived without displaying any clinical symptoms, while none of the mock-vaccinated control animals survived. The CrisBio^®^-derived antigens induced high titers of HA-specific antibodies in the vaccinated poultry, demonstrating hemagglutination inhibition activity against avian H7N1 and human H7N9 viruses. These results suggest that the CrisBio^®^ technology platform has the potential to address major industry challenges associated with producing recombinant influenza subunit vaccines, such as enhancing production yields, scalability, and the speed of development, facilitating the global deployment of highly effective influenza vaccines.

## 1. Introduction

Avian influenza viruses contribute significantly to the emergence of human influenza pandemics. Some of the most notable influenza pandemics in history include the H1N1 Spanish flu of 1918, the H2N2 Asian flu of 1957, the H3N2 Hong Kong flu of 1968, and the H1N1 pandemic of 2009 [1]. Since 2005, avian viruses of the H5 subtype and H7 subtype have caused more than 1000 human deaths and have caused numerous disease outbreaks in domestic poultry and wild birds [2]. These recent avian influenza outbreaks highlight the critical need to enhance preparedness for an impending influenza pandemic. Improved preparedness significantly diminishes the impact of a pandemic. The toll of an influenza pandemic in terms of deaths could be notably higher in developing countries compared to industrialized ones. Vaccines stand out as the most effective measure in reducing both morbidity and mortality. However, their availability during the early stages of a pandemic is unlikely. Additionally, the global supply of vaccines is expected to fall short due to limited production capacity worldwide. Urgent attention is required to bolster pandemic preparedness across all countries, especially in the developing world. 

Currently, influenza vaccines are developed using three distinct technologies: egg-based, cell-based, or recombinant-based methods. Egg-based manufacturing, prevailing for over 70 years, constitutes approximately 82% of commercialized influenza vaccines. This process spans about 6 months, allowing viruses to drift and potentially reducing vaccine efficacy through egg adaptation. Cell-based influenza vaccine production, FDA-approved in 2012, marks the first non-egg-based technology, employing Madin–Darby canine kidney (MDCK) cells instead of fertilized chicken eggs. This approach shortens production time, is not reliant on the egg supply, eliminates the risk of egg-adapted changes, and yields vaccines more akin to the circulating strain, enhancing efficacy. Currently, Flucelvax by Seqirus is the sole FDA-approved cell-based influenza vaccine. Recombinant vaccines, like Flublok developed by Protein Sciences (now part of Sanofi), employ baculovirus vector technology to synthesize the purified virus hemagglutinin (HA) antigen within insect cells. This method, the fastest among current options, can generate vaccine quantities in 6 to 8 weeks, proving advantageous in a pandemic or amid egg shortages. Despite the existence of these vaccine production methods for influenza, the long-term goal is to reduce reliance on egg-based approaches and embrace newer, quicker, and more scalable vaccine technologies for swift responses to novel influenza outbreaks and pandemics.

Baculoviruses, abundant in nature, serve essential ecological roles in the populations of the insects they infect [3]. They do not infect vertebrates, and their viral DNA cannot replicate in mammalian cells, ensuring complete safety from biohazards and posing no risk to operators. Baculoviruses, with a double-stranded DNA genome of approximately 130 kb pairs, can be easily engineered to carry the desired genes, demonstrated by Smith et al. nearly four decades ago when they expressed human β-interferon in insect cells [4]. This marked the inception of the baculovirus–insect cell expression era, leading to the production of thousands of proteins using the Baculovirus Expression Vector System (BEVS). The BEVS presents a simpler, safer, faster, and more easily scalable method for producing recombinant proteins at a lower cost than traditional mammalian cell-based systems. Insect cell systems, mimicking the pattern of mammalian cells and their capacity for cotranslational and posttranslational modifications, have gained popularity in academia and industry for producing the viral structural proteins used in vaccines, therapies, and diagnostic assays to swiftly combat epidemiological emergencies [5].

Among baculoviruses, the *Autographa californica* multiple nuclear polyhedrosis virus (AcMNPV) has been studied extensively and has been implemented for biotechnological purposes [6]. Several commercially susceptible cell lines derived from the *Spodoptera frugiperda* larva (Sf21, Sf9, expresSF+) or *Trichoplusia ni larvae* (BT1-Tn5B1-4, marketed as High Five^TM^) are available. The veterinary vaccines manufactured using this system to prevent classical swine fever or to protect against porcine circovirus PCV-2 have been commercialized. The human vaccines approved by the EMA and FDA, like Cervarix GSK’s bivalent human papillomavirus (HPV 16/18) vaccine against cervical cancer and Flublok’s against influenza, are produced using this technology [7]. 

**Figure 1:**
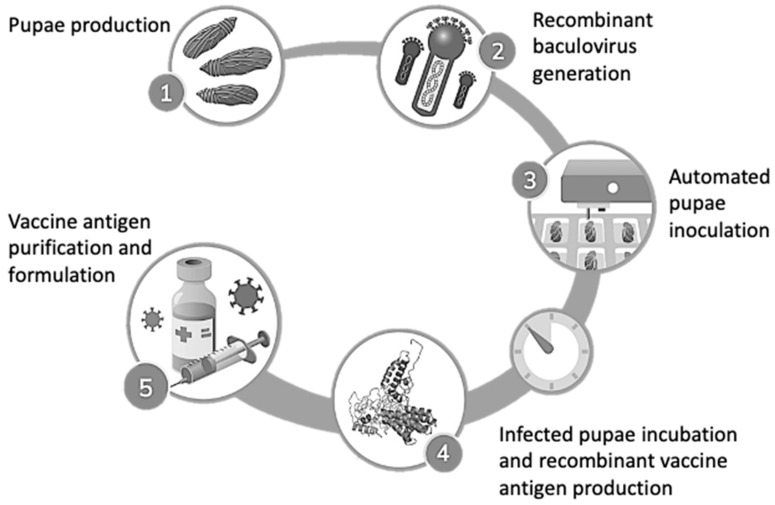
Schematic representation of the manufacturing process of recombinant subunit vaccines with CrisBio^®^ technology.

In the baculovirus expression system, not only cultured cells but also insect larvae and pupae can be used for protein production. Implementing living insect larvae and pupae as single-use biofactories offers a cost-effective means to improve antigen expression levels [8,9]. The CrisBio^®^ technology, combining *T. ni* pupae with recombinant AcMNPV-derived baculoviruses (such as Fatrovax RHDV by Algenex and Fatro), represents a groundbreaking approach. A schematic representation of the manufacturing process of recombinant subunit vaccines with CrisBio^®^ technology is shown in Figure 1. For instance, in influenza, expressing the HA ectodomain fused with specific signals in *T. ni* larvae or *Spodoptera litoralis* larvae resulted in significantly higher expression levels compared to Sf21 cells. These insect-derived HAs induced protective immunity in vaccinated mice [10].

At an industrial scale, insect cells, like other cultured cells, necessitate artificial media and strict environmental sterile conditions in complex and expensive bioreactors. CrisBio^®^ technology offers an efficient alternative by utilizing natural biocapsules (*T. ni* pupae), each housing millions of insect cells in optimal physiological conditions. These pupae can be programmed by genetically modified AcMNPV-derived baculovirus vectors to produce large quantities of any recombinant protein. This technology, tested for dozens of proteins, has shown productivity in the range of milligrams per infected pupa, translating into numerous vaccine doses. This work represents a significant step forward by demonstrating the efficacy of a vaccine antigen produced using this technology in conferring full protection against an influenza pandemic in poultry. The cost-efficiency, simplicity, scalability, and rapid development facilitated by this technology for influenza vaccine production could revolutionize preventive measures during the pandemic situations that could arise from this significant infectious disease.

## 2. Materials and Methods

### 2.1. Cell Culture

The Spodoptera frugiperda Sf21 cell line (Invitrogen, San Diego, CA, USA) was cultured at 28 °C in TNM-FH medium (PAN Biotech GmbH, Aidenbach, Germany). This medium was supplemented with 10% heat-inactivated fetal bovine serum (PAN Biotech GmbH), gentamicin (50 µg/mL) (PAN Biotech GmbH), and an antibiotic/antimycotic solution (Sigma-Aldrich, Burglinton, MA, USA). Cell viability was determined using trypan blue staining, calculating the frequency (%) of living cells relative to the total cell count.

The regulatory Sf9-RVN Glycobac cell line (Sigma-Aldrich, USA) was cultured at 28 °C. In suspension, it was grown in an ESF-921 insect cell culture medium (Expression Systems, Davis, CA, USA), and in adherence it was cultured in an Sf900 III cell culture medium (Gibco, San Diego, CA, USA). Both media were supplemented with gentamicin (50 µg/mL) (PAN Biotech GmbH) and an antibiotic/antimycotic solution (Sigma-Aldrich, USA). The cell viability was assessed through trypan blue staining, determining the frequency (%) of living cells in relation to the total cell count.

### 2.2. Baculovirus Generation

Two recombinant baculoviruses expressing the HA ectodomain antigen from Influenza virus A/H7N1/Italy/2013 were engineered. The resulting sequences were designated as follows:

#### 2.2.1. Mel-H7-His

MKFLVNVALVFMVVYISYIYADKICLGHHAVSNGTKVNTLTERGVEVVNATETVERTNVPRICSKGKRTVDLGQCGLLGTITGPPQCDQFLEFSADLIIERREGSDVCYPGKFVNEEALRQILRESGGIDKEAMGFTYSGIRTNGTTSTCRRSGSSFYAEMKWLLSNTDNAAFPQMTKSYKNTRKDPALIIWGIHHSGSTTEQTKLYGSGNKLITVGSSNYQQSFVPSPGERPQVNGQSGRIDFHWLMLNPNDTVTFSFNGAFIAPDRASFLRGKSMGIQSGVQVDANCEGDCYHSGGTIISNLPFQNINSRAVGKCPRYVKQESLLLATGMKNVPEIPKGRGLFGAIAGFIENGWEGLIDGWYGFRHQNAQGEGTAADYKSTQSAIDQVTGKLNRLIEKTNQQFELIDNEFTEVEKQIGNVINWTRDSMTEVWSYNAELLVAMENQHTIDLTDSEMNKLYERVKRLLRENAEEDGTGCFEIFHKCDDDCMASIRNNTYDHSKYREEAMQNRIQIDPVHHHHHH

#### 2.2.2. Mel-H7-FD4-His

MKFLVNVALVFMVVYISYIYADKICLGHHAVSNGTKVNTLTERGVEVVNATETVERTNVPRICSKGKRTVDLGQCGLLGTITGPPQCDQFLEFSADLIIERREGSDVCYPGKFVNEEALRQILRESGGIDKEAMGFTYSGIRTNGTTSTCRRSGSSFYAEMKWLLSNTDNAAFPQMTKSYKNTRKDPALIIWGIHHSGSTTEQTKLYGSGNKLITVGSSNYQQSFVPSPGERPQVNGQSGRIDFHWLMLNPNDTVTFSFNGAFIAPDRASFLRGKSMGIQSGVQVDANCEGDCYHSGGTIISNLPFQNINSRAVGKCPRYVKQESLLLATGMKNVPEIPKGRGLFGAIAGFIENGWEGLIDGWYGFRHQNAQGEGTAADYKSTQSAIDQVTGKLNRLIEKTNQQFELIDNEFTEVEKQIGNVINWTRDSMTEVWSYNAELLVAMENQHTIDLTDSEMNKLYERVKRLLRENAEEDGTGCFEIFHKCDDDCMASIRNNTYDHSKYREEAMQNRIQIDPVGYIPEAPRDGQAYVRKDGEWVLLSTFLHHHHHH

These encoding sequences were synthesized by GenScript. The codon usage of the HA encoding genes was optimized for expression in insect cells using the OptimumGene™ Codon Optimization algorithm. Both sequences contained flanking regions facilitating their cloning in the TopBac^®^ 3.2 donor plasmid. This plasmid contains an expression cassette that enhances recombinant protein production in insect cells [11].

After obtaining the TB3.2-modified donor plasmids with the H7-His or H7-FD4-His genes, bacmids for generating the baculoviruses were prepared in E. coli DH10Bac bacteria (Invitrogen, San Diego, CA, USA) containing the mini Tn7-replicon. Subsequently, transfection of the bacmids into Sf9-RVN Glycobac cells (Sigma-Aldrich, Burglinton, MA, USA) was conducted, and viral clone selection was carried out through two rounds of plaque cloning. The resulting baculoviruses were titrated using a standard plaque protocol in Sf21 cells (Invitrogen, San Diego, CA, USA), with the virus titer measured in plaque-forming units (pfu).

### 2.3. Pupae Production Process

Insect rearing and pupae production followed the methodology outlined by Escri-bano JM et al. (2020) [8]. Initially, disposable insect rearing boxes were set up with a specific quantity of *T. ni* eggs placed on filter paper. These boxes were then kept in controlled conditions at 21–27 °C and 50–70% humidity for 8 days until pupation occurred in 50–70% of the population.

The eggs hatched and progressed through stages 1 to 5 before entering the pupation phase, typically taking around 10 days. Throughout this period, the insect diet was replaced twice, and the waste produced by the insect larvae was regularly removed.

Upon completion of pupation, a mild alkaline treatment was applied for three minutes to remove the silk surrounding the pupae. Subsequently, the pupae underwent thorough rinsing to eliminate any residual chemicals. Following this, they were dried and automatically placed in disposable plastic trays designed for storage and transportation. Each tray, housing 160 pupae, was RFID-labeled to include information about the pupae’s expiration date.

### 2.4. Infecting Pupae and Expressing Recombinant Proteins

The pupae were inoculated with 10^5^ pfu using an inoculation robot [8]. The infected pupae were then placed in chambers maintaining a controlled temperature of 28 °C and a humidity between 50–70%, where they were incubated for 4 days. Subsequently, the pupae were gathered, frozen, and stored at −20 °C for subsequent downstream processing.

### 2.5. Protein Extraction and Analysis

The *T. ni* pupae containing the recombinant proteins were homogenized in an extraction buffer composed of 20 mM of Na-phosphate at a pH of 7.4, 300 mM of NaCl, a 1% Complete^®^ protease inhibitor cocktail (Roche, Basel, Switzerland), and 20 mM of Imidazole for both proteins.

The subsequent purification steps involved clarification, diafiltration, and ultrafiltration to yield the purified protein. After centrifugation at 15,000× *g* for 15 min, the supernatants underwent initial filtration through Miracloth papers (Calbiochem) followed by depth filtration using a SartoScale Sartoguard GF+ 0.2 µm filter (Sartorius, Goettingen, Germany).

These filtered supernatants were employed for the protein purification via an affinity chromatography using a HisTrap FF Crude 1mL histidine-tagged protein purification column (Cytiva, Marlborough, MA, USA) within an ÄKTA™ Start system (Cytiva). The purified recombinant proteins were eluted from the column using an elution buffer containing 20 mM of Na-phosphate at a pH of 7.4, 300 mM of NaCl, a 1% Complete^®^ protease inhibitor cocktail (Roche, Basel, Switzerland), and 500 mM of Imidazole at a pH of 7.4. The eluate was then diafiltered using a Pierce™ 5 mL Desalting cartridge (Thermo Scientific, Waltham, MA, USA) to remove the Imidazole.

The quantification of the purified recombinant protein was performed via an SDS-PAGE densitometry using a BSA standard curve.

### 2.6. Western Blot

For detecting the recombinant proteins, the total cell or pupae extracts underwent separation via sodium dodecyl sulfate (SDS)-polyacrylamide gel electrophoresis and were transferred to 0.2 µm nitrocellulose membranes (Bio-Rad, Hercules, CA, USA). These membranes were then saturated with 4% of non-fat dried milk in PBS containing 0.1% of Tween 20 (PBST) for 1 h at room temperature. Subsequently, the membranes were incubated with a 1:5000 dilution of anti-His monoclonal antibody (Takara, Kusatsu, Japan) in PBST for 1 hour at room temperature. After two 15 min washes with PBST, the membranes were incubated with a 1:4000 dilution of sheep anti-mouse immunoglobulin G conjugated to horseradish peroxidase (Cytiva). Finally, the membranes underwent two additional 15 min washes, as described above, and were developed using enhanced chemiluminescence.

### 2.7. Virus

The highly pathogenic avian influenza (HPAI) H7N1 strain (A/chicken/Italy/5093/99) used in this study was kindly provided by Dr. Moreno from the Istituto Zooprofilattico Sperimentale della Lombardia e dell’Emilia Romagna (IZSLER, Brescia, Italy) and corresponds to the fifth passage A/chicken/Italy/5093/99. It was propagated in 11-day-old embryonated specific pathogen-free (SPF) chicken eggs, and the allantoic fluid was collected, with 0.45 µM being filtered and stored at −80 °C until use. Subsequently, it was further diluted in PBS to attain a dose of 10^4.5^ embryo lethal dose 50% (ELD_50_) in 0.1 mL (100 µL). The virus was titrated by ten-fold dilutions in phosphate buffer saline (PBS) and then inoculated in SPF eggs following the Reed and Muench method. 

### 2.8. Animals and Experimental Design

Thirty-three specific pathogen-free (SPF) chickens (VALO BioMedia, Salamanca, Spain) were hatched and randomly allocated into three separate negative isolators, each equipped with HEPA-filtered air and maintained under biosafety level 3 (BSL-3) containment conditions at IRTA-CReSA (Bellaterra, Barcelona, Spain). Throughout the experiment, the chickens had ad libitum access to food and water.

In the experimental setup, one isolator (Group 1, n = 11) served as the negative control. The chickens in this group received a subcutaneous sham vaccination with insect extracts infected with an empty baculovirus mixed at a 70:30 ratio with Montanide™ 71 VG ISA.

The chickens in the second isolator (Group 2, n = 11) were vaccinated at 10 days of age with H7-His, followed by a booster vaccination after a 21-day interval. Simultaneously, in the third isolator (Group 3, n = 11), the chickens were vaccinated at 10 days of age with H7-FD4-His, and a similar 21-day interval booster vaccination was administered. The animals from Groups 2 and 3 received a subcutaneous vaccination of 0.2 mL/animal of PBS containing 25 µg of the corresponding H7 antigen mixed at a 70:30 ratio with Montanide™ 71 VG ISA (Seppic, Courbevoie, France).

Subsequently, all chickens (Groups 1–3) were intranasally inoculated with 100 µL of diluted infectious allantoic fluid containing 10^4.5^ ELD_50_ H7N1 HPAIV. From two days before the challenge onwards, all chickens were monitored daily for flu-like clinical signs. The experiment concluded at 10 days post-infection, at which point all remaining chickens were euthanized. Samples from oropharyngeal swabs (OS) and cloacal swabs (CS) were collected at 1, 2, 3, 5, 7, and 9 (0, 2, 4, 6, and 10) days post-infection (dpi). Additionally, the blood samples for obtaining sera were collected from all individuals at the pre-challenge time-point, the 33rd post-vaccination day (PVD), and at 10 dpi. All gathered samples were stored at −80 °C until their use.

### 2.9. Disease Monitoring

Daily monitoring for clinical signs aligned with the World Organization for Animal Health (OIE) guidelines [12] and involved a semi-quantitative scoring system: healthy (0), sick (1), severely sick (2), and moribund or dead (3). The chickens exhibiting one of the potential flu-like clinical signs were categorized as sick (1), while those manifesting two or more signs were classified as severely sick (2) and were euthanized via intravenous administration of sodium pentobarbital at a dose of 100 mg/kg (Dolethal^®^, Vétoquinol, Madrid, Spain). The potential flu-like clinical signs included respiratory involvement, depression, diarrhea, facial or head edema, cyanosis of exposed skin or wattles, and nervous signs.

### 2.10. Real-Time Quantitative PCR (RT-qPCR)

All swab samples (OS and CS) underwent examination for viral RNA quantification. The viral RNA was extracted using the NucleoSpin RNA isolation kit (Macherey-Nagel, Düren, Germany), and a 99 base pair (bp) fragment of the M gene was amplified as previously described [13] using the Fast7500 equipment (Applied Biosystems, Foster City, CA, USA). The detection limit of this technique was 0.84 log10 GEC/mL viral RNA copies per sample.

### 2.11. H7-Specific Enzyme-Linked Immunosorbent Assays (ELISA)

The ELISA plates were coated with 2 µg/mL of H7 antigen acquired from Sino Biological (cat no. 40169-V08H1) (Sino Biologicals, Eschborn, Germany), corresponding to the A/turkey/Italy/4602/99 strain, diluted in 50 mM of sodium bicarbonate buffer, and incubated overnight at 4 °C. Subsequently, the plates were blocked for 1 h at 37 °C with a 100 μL/well of 3% BSA/PBS 1x. The diluted 1:100 chicken sera (50 μL/well) were then incubated for 1 hour at 37 °C. The plates underwent four washes with 0.5% Tween20/PBS 1x and were subsequently incubated for 45 min at 37 °C with a diluted 1:50,000 HRP-conjugated goat anti-chicken IgY (ab6877, Abcam, Cambridge, UK) (50 μL/well). After four more washing steps, a 50 µL/well of 3, 3′, 5, 5′-tetramethylbenzidine (TMB) substrate solution was dispensed. The reaction was stopped with 1 N of H_2_SO_4_ after 10 min and protected from light. Finally, the plates were read at an optical density (OD) of 450 nm. The samples were evaluated in duplicates. Each plate included positive H7N1 and negative control anti-serums (GD-Animal Health Service, Deventer, The Netherlands). 

### 2.12. Hemagglutination Inhibition (HI) Assay

The HI tests were conducted following the standard protocol for the diagnosis of avian influenza from the WOAH-OIE (Manual of Diagnostic Tests and Vaccines for Terrestrial Animals 2019). Briefly, the serial diluted sera were incubated with 4 HA units of the H7N1 antigen (cat nº VLDIA098) (Royal GD-Animal Health, Deventer, The Netherlands) and the H7N9 antigen f A/Anhui/1/2013 (cat nº NIBRG-268) (NIBSC, South Mimms, UK). The sera–viral antigen mixture was further incubated with 1% of chicken red blood cells (cRBCs). Positive H7N1 and negative control anti-serums (GD-Animal Health Service, Deventer, The Netherlands) were included in each plate. All samples were assayed in duplicate. The HI titers were defined as the reciprocal sera dilution, where complete inhibition of hemagglutination was observed.

### 2.13. Statistical Analysis

The significance between survival curves was determined using the Kaplan–Meier survival analysis with the log-rank (Mantel–Cox) test. A normality assessment was conducted using a Shapiro–Wilk test followed by the Student’s *t*-test (for the normally distributed data) or Wilcoxon test (for non-normally distributed data). The statistically significant differences among the groups were indicated when *p* values were less than 0.05, denoted as follows: *p* < 0.05 (*), *p* < 0.01 (**), *p* < 0.001 (***), and *p* < 0.0001 (****). The R 4.0.4 statistical software was utilized for developing all the statistical analyses (http://cran.r-project.org/).

## 3. Results

### 3.1. Production of Monomeric and Trimeric Versions of HA Using CrisBio^®^ Technology

**Figure 2:**
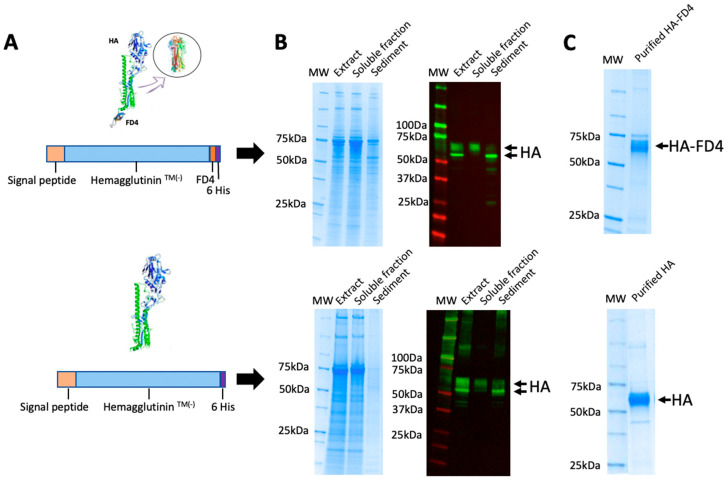
(**A**) Schematic representation of the genetic constructs expressed by baculovirus vectors with CrisBio^®^ technology. (**B**) Coomassie blue staining and a Western blot of the pupae-derived extracts obtained with the different baculovirus vectors, showing the total as well as the soluble and insoluble fractions. (**C**) Affinity-purified recombinant HA proteins derived from pupae extracts.

To investigate the feasibility of expressing the HA protein (A/chicken/Italy/5093/99; H7N1) within *T.ni* pupae as living biofactories, a newly synthesized cDNA fragment encoding the HA gene lacking the transmembrane domain, deemed irrelevant for the protective immune response, was designed. The codon usage was adapted for expression in insect cells. Additionally, the gene was altered by incorporating a melittin signal peptide and a C-terminal hexahistidine (6×His) tag (H7-His). A second construct was generated, incorporating the trimerization domain FD4 upstream to the His tag (H7-FD4-His) (Figure 2A). These constructs were inserted into an expression cassette, TopBac^®^, to generate recombinant baculoviruses.

The resulting baculoviruses, named BacHA and BacHAFD4, were utilized to infect *T. ni* pupae under various infection conditions to optimize productivity. We employed doses of 10^3^, 10^4^, and 10^5^ pfu, collecting the pupae at 3, 4, or 5 days post-infection. After three independent experiments, the optimal conditions for the recombinant protein production in both baculoviruses were identified as a dose of 10^4^ and 4 days of infection. The HA protein showed two forms with differing molecular weights, presumably attributed to distinct glycosylation statuses. Interestingly, the higher molecular weight HA proteins induced by both baculoviruses (65–75 kDa) exhibited solubility, whereas the lower molecular weight protein bands (approximately 60 kDa) were notably insoluble (Figure 2B). These data imply that proper conformation and posttranslational modifications influence protein solubility in pupae cells.

The soluble extracts obtained from the pupae infected under optimal conditions were employed for purifying the two HA protein versions via affinity chromatography, facilitated by the His tag in both constructs. Remarkably, we achieved purification levels exceeding 70% for the HA proteins in a single step (Figure 2C). The productivity of the HAFD4 and HA proteins in the crude pupae extracts, as well as their yields after purification under the established experimental conditions, were found to be highly similar.

### 3.2. Protection of Vaccinated Poultry against a Highly Virulent H7N1 Influenza Virus

**Figure 3:**
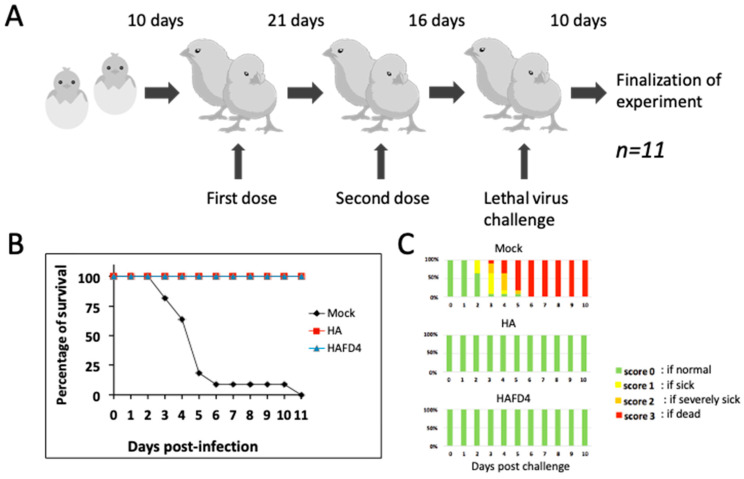
(**A**) Schematic representation of the chicken vaccination procedure and challenge with the highly pathogenic virus, H7N1 strain (A/chicken/Italy/5093/99). (**B**) Number of poultry belonging to the different experimental groups that survived at different days post-challenge with the virulent influenza virus. (**C**) Percentage of the poultry belonging to the experimental groups showing different pathology scores.

To assess the protective efficacy in poultry of the two HA proteins derived from the CrisBio^®^ technology, we immunized two groups, each comprising 11 birds, with each HA protein formulated with a mineral oil adjuvant. The initial dose of 20 μg was administered at 10 days of age, followed by a second boost dose 21 days later. Concurrently, a control group of the same size was immunized using the same protocol but with extracts obtained from pupae infected with a wild-type TopBac^®^-modified baculovirus vector and subjected to identical purification procedures. Subsequently, after 16 days from the second dose, all three groups were challenged with the highly virulent H7N1 avian influenza virus (A/chicken/Italy/5093/99). For the following 11 days after challenge, the health status of all vaccinated poultry from the three groups were closely monitored (Figure 3A).

All poultry from the groups immunized with the CrisBio^®^-derived HA proteins survived the challenge infection (Figure 3B) without exhibiting any clinical symptoms of Highly Pathogenic Avian Influenza (HPAI disease, as per the scoring system by the World Organization for Animal Health). In contrast, the majority of mock-vaccinated control poultry perished by day 6, and none survived beyond day 11 post-challenge. The control group poultry displayed severe clinical symptoms between days 3 and 4 post-challenge, preceding their demise (Figure 3C).

**Figure 4:**
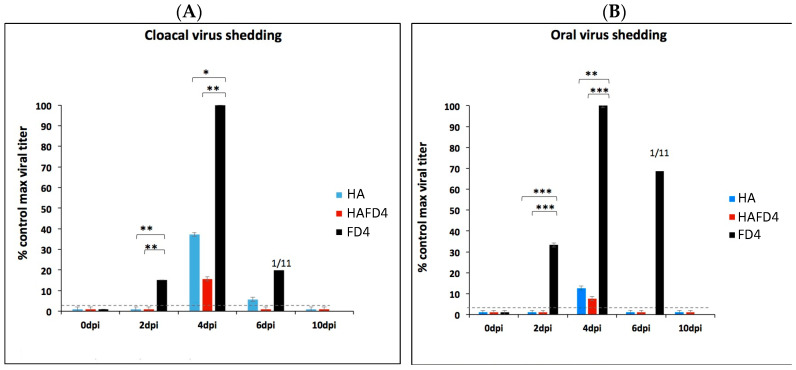
(**A**) Cloacal and (**B**) oral virus shedding of the chickens belonging to the experimental groups at different days post-challenge with the highly pathogenic virus, H7N1 strain (A/chicken/Italy/5093/99). Student *t*-test: * *p* < 0.05, ** *p* < 0.01, *** *p* < 0.001.

To explore another aspect of the protection, we investigated cloacal and oral virus shedding in the three poultry groups. While viral shedding from both cloacal and oral routes was detectable in the control group as early as day 2 post-infection, the vaccinated poultry, regardless of the HA proteins used, exhibited reduced virus shedding by day 4 (Figure 4). By day 6 post-infection, cloacal virus shedding was observed in the HA-vaccinated poultry, whereas the HAFD4-vaccinated poultry showed no detectable cloacal shedding. Notably, no virus was detected in the oral cavities of both groups vaccinated with the recombinant proteins. By day 10, no virus shedding was detectable in either the HA- or HAFD4-vaccinated poultry groups. In contrast, the maximum viral shedding in the control group occurred at day 4, both in the cloacal and oral cavities, just before the majority of poultry succumbed to the infection (Figure 4).

This vaccination experiment clearly demonstrates that both the CrisBio^®^-derived HA proteins offered complete protection against a highly virulent influenza virus pandemic that resulted in a 100% mortality rate among the mock-immunized poultry group. Interestingly, no discernible differences were observed between using the monomeric or trimeric HA proteins in terms of providing protection (Figure 3B) or preventing clinical symptoms (Figure 3C). However, it appears that the trimeric HA protein significantly reduced oral and cloacal virus shedding to a greater extent than the monomeric HA protein compared to the control group (Figure 4A,B). This suggests that the trimeric HA protein exhibits a more pronounced protective effect by reducing the spread and viral load, particularly in terms of limiting the viral shedding from the oral and cloacal routes.

### 3.3. Antibody Response in Vaccinated Poultry

**Figure 5:**
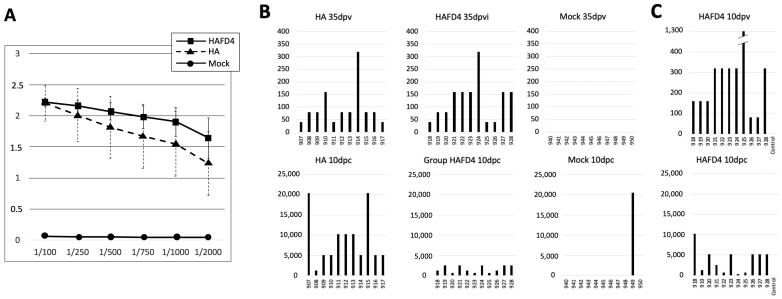
Antibody response raised in chickens belonging to the different experimental groups before and after the virulent influenza virus challenge with the highly pathogenic virus, H7N1 strain (A/chicken/Italy/5093/99). (**A**) Neutralizing antibodies in poultry sera at the day of challenge. (**B**) Homologous avian virus hemagglutinin inhibition antibody titers in sera before and after virus challenge. (**C**) Heterologous human hemagglutinin inhibition antibody titers against a related human isolated virus, A/Anhui/1/2013 H7N9, in sera before and after virus challenge.

To assess the humoral immune response elicited in the HA- and HAFD4-vaccinated poultry groups in comparison to the mock-vaccinated control group, we evaluated the IgY antibody titers using an ELISA test. The sera that recovered 35 days after the initial vaccination dose were subjected to several dilutions for ELISA analysis just before the virus challenge. The results revealed that all poultry vaccinated with both CrisBio^®^-derived HA proteins developed high IgY antibody titers (>1:2000) against a homologous HA7 protein (Figure 5A). While both HA and HAFD4 vaccines effectively induced a humoral response against the homologous H7, the HAFD4 appeared more immunogenic than the HA, eliciting an increased antibody response compared to the HA.

Furthermore, to delve deeper into the humoral immune response post-vaccination, we determined homologous avian H7- and heterologous human H7-specific antibody titers through an HI test. Assessing the functionality of these antibodies, potentially explaining the protection conferred by the CrisBio^®^-derived HA proteins, we analyzed the hemagglutination inhibition against an avian H7N1 virus and a closely related human isolated virus, A/Anhui/1/2013 H7N9. The sera collected from the vaccinated poultry at day 35 after the initial immunization dose and at day 10 post-virus challenge were used in this analysis. A notable difference in functionality was evident between the sera from poultry vaccinated with CrisBio^®^-derived HA proteins and those from the mock-vaccinated group (Figure 5B). Both the HA- and HAFD4-vaccinated animal sera consistently exhibited HI titers (>1:40) against the homologous virus. Interestingly, these same sera displayed notably high HI titers against the heterologous H7N9 human virus, in some cases surpassing the titers against the avian virus (Figure 5B). Generally, the sera from the HAFD4-vaccinated poultry exhibited higher HI titers than those from the HA-vaccinated group (Figure 5B). The slight increase in the HI titers observed with the HAFD4 construct potentially explains the reduction in the HI titers after the virus challenge and the subsequent decrease in virus shedding in this group compared to the HA-vaccinated group.

These findings unequivocally demonstrate that the proteins produced via CrisBio^®^ technology possess the necessary conformational properties to induce functional antibodies in vaccinated poultry. Intriguingly, antibodies generated against avian H7N1-derived proteins exhibited equal functionality against the human H7N9 virus (Figure 5C), displaying even higher HI titers against the human virus compared to the avian virus H7N1.

## 4. Discussion

Influenza vaccine production encounters several challenges, including the substantial need for embryonated eggs, antigenic alterations during egg adaptation, and difficulties in achieving high virus yields [14]. Furthermore, swiftly adapting vaccines to circulating viruses poses a significant hurdle. Recombinant subunit vaccines offer a promising solution to these issues. Typically, the yield of total viral protein from 200 embryonated eggs averages around 20–40 mg [15]. Consequently, the large-scale production of inactivated vaccines is notably challenging and costly.

During an influenza pandemic, alternative vaccine manufacturing processes become crucial. Many potential pandemic strains stem from birds, significantly affecting vaccine production if hens laying the required eggs are infected. This could result in reduced egg production or, if the virus is lethal to chicken embryos, it may limit the antigen production. To address these challenges, continual development and testing of new platforms that do not rely on eggs or live influenza virus and are more resilient to such problems remain ongoing. One such example is RNA-based vaccines, demonstrating promising results in expressing influenza proteins, especially in animals [16]. However, the duration of immunity using this type of vaccine remains uncertain, and there are limitations regarding the administered dose. Traditional protein-based vaccines remain predominant in the realm of recombinant subunit vaccines for infectious diseases due to factors such as safety, efficacy, and the potential for long-term protection, which can be modulated by using adjuvants.

Various alternatives exist to produce correctly glycosylated and folded vaccine antigens capable of immunizing animals or humans. The genetic modification of mammalian cells, commonly used to express recombinant monoclonal antibodies, is not ideal for producing recombinant subunit influenza vaccines due to the technology’s inherent lengthy development times, an inability to address the demand for antigen sequence updates in seasonal vaccines, and limited agility in pandemic situations. In contrast, the Baculovirus Expression Vector System (BEVS) is more suitable due to its rapid development times and high productivity. The first vaccine produced in insect cells (Flublok; Protein Sciences–Sanofi) was approved in 2013. However, using cultured cells in recombinant biologics manufacturing has limitations in scalability and cost-efficiency, particularly in low-income economies. The key advantage of the baculovirus expression system lies in its ability to generate substantial amounts of recombinant proteins within a relatively short timeframe. Previous studies have reported high yields of viral HA protein using baculovirus–insect cell systems [17]. 

Recognizing these bottlenecks, technologies utilizing living organisms as biofactories were developed to improve development times, cost-efficiency, and scalability by simplifying the manufacturing processes compared to cultivated cells in bioreactors. One such technology, the transient expression of antigens in Nicotiana benthamiana plants, has emerged as a mature platform for producing influenza vaccines. Ward and colleagues recently reported two efficacy studies, the first randomized phase 3 trials of a plant-derived quadrivalent influenza vaccine [18]. The field of plant-derived vaccines has seen substantial growth since the demonstration in 1992 that viral proteins could be expressed in plants [19]. However, a concern with plant-based production during pandemics is the time required for material generation, involving the generation of the Agrobacterium vector and the subsequent plant growth for transfection.

Another platform highly explored for experimental vaccine production involves insect larvae or pupae [8,9,11,20]. Insects, growing in large numbers in simple infrastructures in short periods under captivity, offer remarkable potential. Several insect species are mass-produced today for obtaining proteins and fats used in animal feed [21,22]. Hence, the technology for obtaining significant insect quantities in laboratories is well-established. Currently, two insects, *Bombyx mori* and *Trichoplusia ni* Lepidoptera, are utilized for manufacturing therapeutic or vaccine products using baculovirus vectors. Products from both insects have favorably passed regulatory processes (*Bombyx mori* larvae, Virbagen^®^ Omega in 2004; and *Trichoplusia ni* pupae, Fatrovax^®^ RHDV in 2021). While *B. mori* uses a specific baculovirus vector called *Bombyx mori* nucleopolyhedrovirus (BmNPV) for the protein manufacturing [23,24], recombinant proteins in *T. ni* employ the conventional *Autographa californica* multipolyhedrovirus vector (AcMNPV) [8,9,20], similar to that used in SF9 or Hi-5 cultured insect cells. This indicates that any available baculovirus can be directly used for production in the CrisBio^®^ technology. In our work, we employed an enhanced baculovirus called TopBac^®^, significantly boosting recombinant protein productivity in cells and insects [8,9,10]. By combining TopBac^®^ and CrisBio^®^, we achieved an HA protein production of approximately 75 mg/L of pupae extract, purified to >70% in a single affinity chromatography step. Productivity differences based on the HA sequences were observed, yet these quantities were notably higher than those in cultured insect cells or eggs. These production rates represent ~4 vaccine doses of 20 μg of recombinant HA per infected pupae. An extensive cost analysis of manufacturing concluded that producing recombinant biologics in CrisBio^®^ might account for 30% of COGs and requires four times less investment in a cGMP factory compared to cultured insect cells.

Prior research has confirmed that the baculovirus expression system correctly glycosylates the HA protein, ensuring the presence of N-linked oligosaccharide side chains in the HA1 region [25]. Our earlier work comparing the glycosylation patterns and abundance of different glycosylation forms between insect cells and insects confirmed this. Notably, our experimental vaccination study in poultry with two versions of the CrisBio^®^-derived recombinant HA protein indicated that the expressed proteins maintained their immunogenicity, indicating accurate protein modifications and folding.

Collectively, these findings suggest that the HA proteins expressed in *T. ni* pupae, either as C-terminus fusion proteins with the FD4 trimerization domain or with a His-Tag sequence alone, can be produced in significant quantities in biochemically active forms. Despite the complexity of pupae extracts, easy purification was achieved, eliminating potential adverse reactions in vaccine recipients as these extracts lack egg contaminants such as ovoalbumin, a common allergen in humans. Moreover, influenza virus inactivation or organic extraction procedures were unnecessary, mitigating potential denaturing effects and safety concerns arising from residual toxic chemicals in the vaccine.

The integration of CrisBio^®^ recombinant technology in influenza vaccine production, not only for poultry but potentially for other animal species or humans, could help mitigate the drawbacks of current egg-based or insect cell-based production methods. CrisBio^®^ technology can be swiftly adapted to the large-scale manufacturing of various HA antigens or in combination with other influenza antigens such as NA (some experimental long-lasting immunity vaccines are under evaluation [26]), enabling the deployment of seasonal and pandemic influenza vaccines within approximately three months, a marked improvement over the 6 to 8 months required using fertile egg technology. Therefore, CrisBio^®^ presents a compelling alternative to current egg-based inactivated vaccines or the conventional production of recombinant HA-based vaccines using insect cells, particularly in terms of development timelines and the rapid large-scale manufacturing of seasonal vaccines. In this study, we showcased the CrisBio^®^ platform, which yielded unprecedented production yields per liter of pupae extract following a single step of chromatographic affinity purification. 

In conclusion, this work marks an initial effort to produce an influenza pandemic vaccine on the CrisBio^®^ platform, affirming that the expressed HA protein functionally preserved immunogenicity, preventing death and clinical symptoms in vaccinated poultry challenged with a highly virulent virus that killed all animals in the control group. These experiments pave the way for exploring the CrisBio^®^ platform’s potential in developing subunit influenza vaccines for humans and animals.

## Data Availability

All data created in this study have already been shown in the manuscript. No more new data is available.
